# 4S Peak Filling – baseline estimation by iterative mean suppression

**DOI:** 10.1016/j.mex.2015.02.009

**Published:** 2015-02-21

**Authors:** Kristian Hovde Liland

**Affiliations:** a Norwegian University of Life Sciences, 1430 Ås, Norway; b Nofima – Norwegian Institute of Food, Fisheries and Aquaculture Research, 1432 Ås, Norway

**Keywords:** Baseline estimation by iterative mean suppression, Baseline estimation, Smoothing, Subsampling, Moving window, Noise, Interpolation

## Abstract

A novel baseline estimation procedure building on previously published works is presented.

• The core of the estimation is an iterative spectrum suppression consisting of a moving window minimum replacement (adapted from Friedrichs [1]).
• Four, easily understandable, parameters control placement of the baseline relative to the noise band around the signal (adapted from Eilers [2]) and the flexibility in different situations.
• The method is especially suited for non-linear baselines with local variations and for resolving peak clusters in qualitative analyses.

## Graphical abstract

**Figure:**
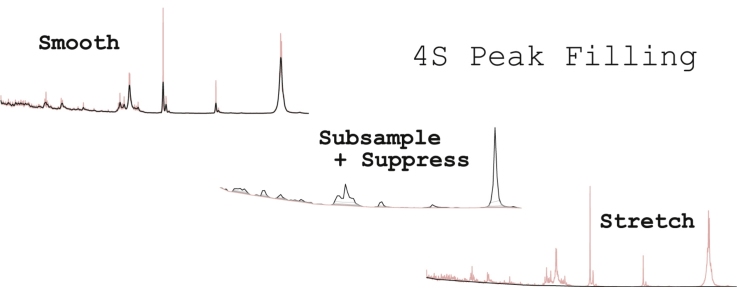

## Methods

There are four main ingredients in the 4S Peak Filling baseline estimation procedure. These are described below, one S at a time. Supporting the descriptions are illustrating figures with spectra from matrix assisted laser desorption/ionisation time-of-flight (MALDI-TOF) [3] and laser induced breakdown spectroscopy (LIBS) [4]. The former is included to illustrate the effects of the different steps of the algorithm, while the latter presents a problem where the proposed method handles a challenging baseline estimation where other tested methods fail. An R [5] implementation with section numbers corresponding to this article is freely available in the ‘baseline’ package in the Comprehensive R Archive Network repository.

## Smoothing

**Fig. 1:**
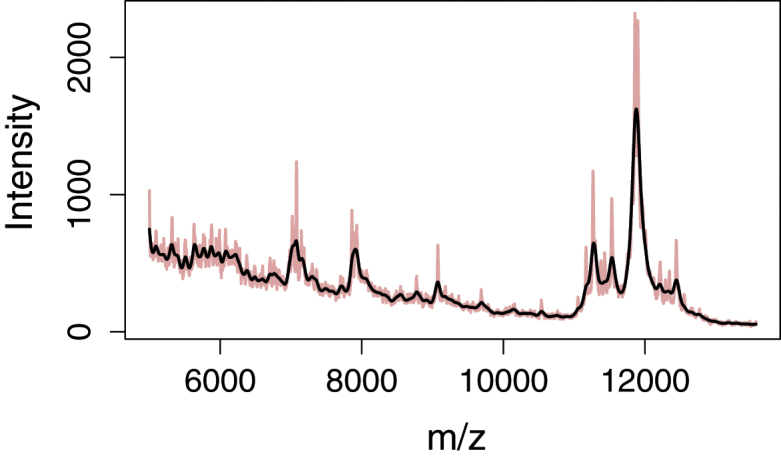
Smoothing of the original MALDI-TOF spectrum by Whittaker (*λ* = 10^4^).

Before baseline estimation is performed on the spectra, they are smoothed by applying a penalty on their second derivative; see Fig. 1 and Section S1 in the R code. This is achieved using the Whittaker smoother as described in Eilers’ article on a perfect smoother [6]. Other smoothers may have the desired effects, but the Whittaker smoother is a quick and well-tested basis for smoothing and baseline estimation.

There are two important effects of applying a smoother. First of all, it limits spurious noise peaks that are of no interest in the baseline estimation. More importantly it gives the user the possibility of centring the baseline in the noise band around the signal spectrum by adjusting the smoothing parameter, thus giving a more realistic zero signal for most types of spectra. It does not matter that real peaks may be shrunk severely in the smoothing as this does not harm the baseline estimation. If no smoothing is performed, the baseline will be placed in the bottom of the noise band.

## Subsampling

**Fig. 2:**
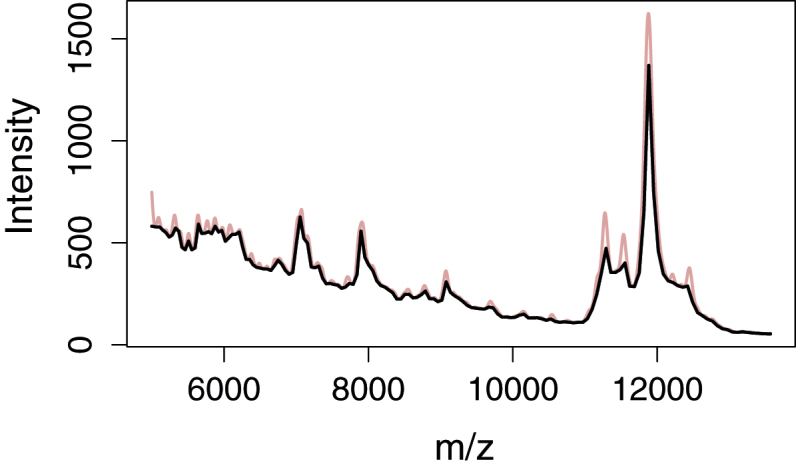
Subsampling of smoothed MALDI-TOF spectrum, reducing resolution from 4000 to 150 *m*/*z* values.

Instead of estimating the baselines directly on the spectra, a simple binning is performed first; see Fig. 2 and Section S2 in the R code. The number of bins is chosen by the user, and the minimum value in each bin is used as a local representative of the spectrum. The subsampling serves two goals. Firstly, it increases the efficiency of the algorithm with regard to the number of local windows it will work in, thus reducing the number of iterations needed to suppress the baseline. Secondly, it simplifies the shape of the spectrum while retaining the basic shape of the baseline.

If the baseline needs to be flexible or very steep, e.g. because of dominating fluorescence in Raman spectroscopy, a high number of bins is needed, while a more linear and flat baseline can be represented by very few bins. A good starting point is to have one bin per 10 spectrum values and then adjust the number according to visual inspection or subsequent analysis performance. Too many bins may cause a baseline that rises into peaks, while too few bins may cause the baseline to “detach” from the spectrum (too low estimate) if the true baseline is highly concave. For spectra having regions of both characteristics, bin widths may need to be varied along the spectra (see Fig. 6).

## Suppression

**Fig. 3:**
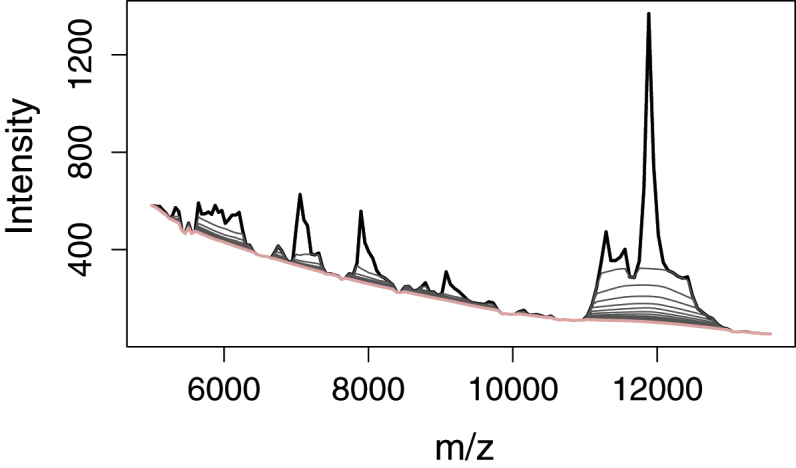
Iterative suppression of baseline from smoothed, subsampled MALDI-TOF spectrum. A window width of ±15 points and 20 iterations was used.

The main part of the algorithm is the iterative suppression; see Fig. 3 and Section S3 in the R code. A window is moved along each spectrum similar to the median window method [1]. There are four features that set the strategies apart. Firstly, the minimum of the current value and the mean value in the window is used instead of the median value. A well-chosen window width and a corresponding number of buckets will ensure that the minimum is sufficiently low but not below the perceived baseline.

The second feature is that the current spectrum is updated for each move of the window instead of estimating all minima before updating the spectrum. The effect is a quicker convergence, and a directional effect. The latter means that the window has to be moved in both directions, first sliding it to the right and then back again, to avoid a biased estimation around peaks.

The third feature is that the window is shrunken logarithmically for each completed window movement right and left, ending up at a width of ±1 wavelength/mass. By this shrinking we increase the effectiveness by allowing wide windows in the first iterations while reducing the chance of ending up with a too low baseline.

The last feature is that the window is symmetrically shrunk when close to the ends of the spectra. This is done to avoid too low minima when the baseline is steep toward the ends, which can be the case for spectra like those of MALDI-TOF or Raman spectra with dominating fluorescence.

The number of passes the window moves right and left over the spectra is a user chosen parameter. This parameter is tightly connected with the number of bins and affects the baseline in a similar manner. Too few iterations will lead to a baseline raising into the peaks. If the baseline is relatively flat, too many iterations will not harm the estimation. But if the true baseline is highly concave, too many iterations may have the same baseline “detaching” effect where the baseline is estimated too low. A possible extension of the algorithm could be to also include a convergence criterion to stop the iterations early when changes are small.

Aiming for a baseline having little flexibility and interpreting the area around 12,000 *m*/*z* in the MALDI-TOF spectrum as a peak cluster rather than a rise in the baseline lead to the choice of a wide (relative to the 150 bins) window width (±15 points). The large width of the mentioned peak cluster meant that a high number of iterations were also needed (20). A starting point when adapting the parameters to a new set of spectra is to choose a window width approximately covering the width of the widest peak and 10 iterations before starting to adjust the parameters.

## Stretching

**Fig. 4:**
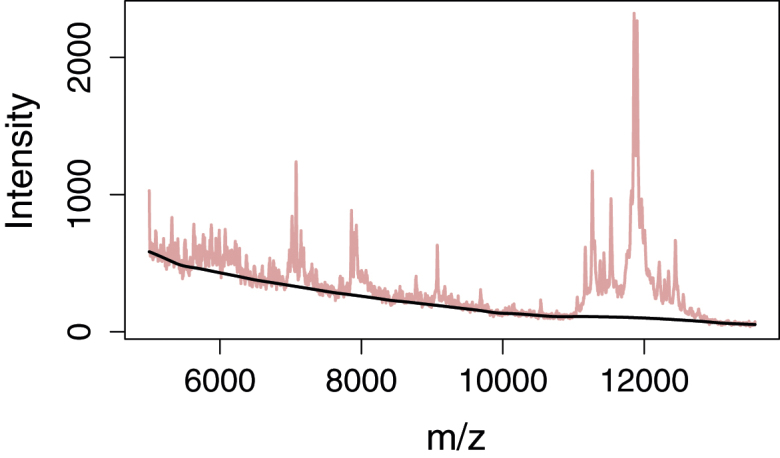
Baseline stretched by smoothing splines after smoothing, subsampling and suppression of MALDI-TOF spectrum.

The final stage of the algorithm consists of interpolating the estimated baseline back to full spectrum length; see Fig. 4 and Section S4 in the R code. Because of the choice of minimum values, we can place the estimated baseline at the centre points of the buckets and find the remaining values using simple, linear interpolation for speed and robustness or a set of properly constrained smoothing splines for better smoothness.

## Subtraction

**Fig. 5:**
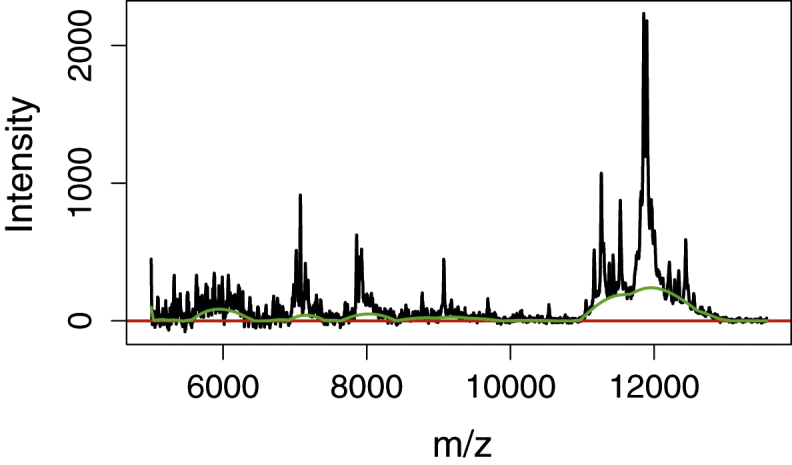
MALDI-TOF spectrum corrected by the estimated baseline. An alternative baseline is indicated which follows the shape of the peak cluster around 12,000 *m*/*z*.

If the baseline estimation is used for baseline correction, we have to subtract the final baseline from the original spectrum as has been done in Fig. 5. We observe that the zero line has been well centred in the noise band. The baseline was chosen to be rigid enough to retain all the small peaks, e.g. around 7000 and 8000 *m*/*z*. By using more buckets and a smaller window width it is possible to place the baseline in the peak cluster around 12,000 *m*/*z* so that the peaks are better resolved as indicated with the curved line in Fig. 5. If more localised control of the baseline flexibility is needed one has to resolve use varying bin widths along the spectra.

**Fig. 6:**
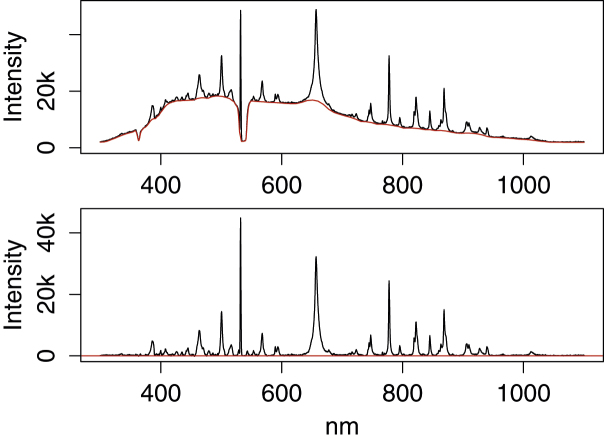
LIBS spectrum corrected using 4S Peak Filling with varying baseline interval widths to handle baseline dips around 360 nm and 530 nm and the steep ridge around 400 nm (no smoothing, ±3 window width, and 2 iterations).

A vastly more challenging baseline can be found in LIBS spectra as shown in Fig. 6. These contain dips in the baseline that are impossible to adapt to without also having a baseline that raises high into the peaks. We tested the asymmetric least squares (ALS) baseline estimation, which is a modified Whittaker smoother, and several other methods with consistently bad results. For instance, if ALS is used with high enough penalty on the second derivative and low enough weight on positive residuals (see Ref. [2]) to give a flat baseline under the peaks, false peaks will appear around the baseline dips after baseline subtraction.

With the proposed method this can be overcome by customizing the subsampling, as proposed in Ref. [7]. Using narrow bins around the baseline dips at 360 nm and 530 nm, wide bins where the baseline looks relatively flat and medium bin widths where the baseline is steep around 400 nm we get the estimation and correction shown in the upper and lower part of Fig. 6. As long as there is not too much shift in the baseline dips from spectrum to spectrum, the chosen bin widths can be used for all spectra in the data set.

## Additional information

The described baseline estimation is implemented in the R package ‘baseline’ in the CRAN repository (http://cran.r-project.org) under the name *fillPeaks*. The four parameters mentioned above are summarized in Table 1.

## Figures and Tables

**Table 1:** Summary of the parameters controlling the baseline estimation.

Description	R name	Suggested starting value
Second derivative penalty for smoothing.	lambda	Centred in noise band: 4 Below spectra: 0
Number of buckets for subsampling or a vector of start/end points of buckets.	int	1/10 of the number of wavelengths/masses/points
Initial half width of windows used for suppression.	hwi	Half the width of the widest peak (full width = centre point ± hwi)
Number of iterations for suppression.	it	10
